# Self-Association of Okadaic Acid: Structural and Pharmacological Significance

**DOI:** 10.3390/md11061866

**Published:** 2013-05-29

**Authors:** Patricia G. Cruz, Manuel Norte, Alberto Hernández Creus, José J. Fernández, Antonio Hernández Daranas

**Affiliations:** 1 University Institute for Bio-Organic Chemistry “Antonio González” (IUBO), University of La Laguna (ULL), Astrofísico Francisco Sánchez 2, 38206 La Laguna, Tenerife, Spain; E-Mails: patgecruz@hotmail.com (P.G.C.); mnorte@ull.es (M.N.); 2 Pharma Mar, Avda. de los Reyes, 1 P.I. La Mina-Norte, Colmenar Viejo, Madrid 28770, Spain; 3 Department of Organic Chemistry, University of La Laguna (ULL), Astrofísico Francisco Sánchez 1, 38206 La Laguna, Tenerife, Spain; 4 Department of Physical Chemistry, University Institute of Materials and Nanotechnology, University of La Laguna (ULL), Astrofísico Francisco Sánchez 1, 38206 La Laguna, Tenerife, Spain; E-Mail: ahcreus@ull.es; 5 Department of Chemical Engineering and Pharmaceutical Technology, University of La Laguna (ULL), Astrofísico Francisco Sánchez 1, 38206 La Laguna, Tenerife, Spain

**Keywords:** okadaic acid, protein phosphatase, nuclear magnetic resonance, scanning tunneling microscopy

## Abstract

Okadaic acid (OA) has been an invaluable pharmacological tool in the study of cellular signaling. The great affinity of this polyether for its targets together with its high specificity to inhibit certain protein phosphatases enables the differential study of these proteins. Crystallographic structures of protein phosphatases in complex with OA show a 1:1 protein to toxin ratio. Nevertheless, it has been found that OA is able to self-associate under certain conditions although very little is known about the importance of this phenomenon. Here we review the available knowledge on the latter topic and we report on the existence of an unusual self-associated tetrameric form. The structure of these oligomers is proposed based on spectroscopic data and molecular modeling calculations.

## 1. Introduction

Okadaic acid (OA) is well known for being the main culprit of the diarrhetic shellfish poisoning, also known as DSP syndrome, a phenomena with important consequences for the human health and the fishery industry; but it has also been an invaluable pharmacological tool in the study of cellular signaling [1,2]. OA was originally isolated from the black sponge *Halichondria okadai* by a Japanese pharmaceutical company, but it was later discovered that marine dinoflagellates of the *Dinophysis* and *Prorocentrum* genera were its genuine originator [3]. The structures of the *o*-bromobenzyl ester of OA as well as that of its episulfide, acanthifolicin, were originally determined by X-ray crystallography [4,5]. Subsequently, the conformation of OA in solution was also elucidated by NMR spectroscopy in two different studies that found a major similarity with that obtained for the solid-state structure [6,7]. Afterwards, in the late 1980s, a study of the cellular regulatory mechanisms underpinning muscle contraction produced by OA showed that it was mediated by inhibition of the phosphatase that dephosphorylates myosin light chains [8,9]. As part of this research, a seminal work by Bialojan and Takai, they compared the effects of OA helped to distinguish between different protein phosphatases. This result was considered a milestone in cell signaling research [10]. Consequently, OA is probably the best known phosphatase inhibitor and has been profusely used to study the cellular role of these proteins [11]. Due to its high but different affinity for protein phosphatases type 1 (10–100 nM) and type 2A (0.1–1 nM), OA has been used in several structure-activity studies looking for selective phosphatase inhibition [12,13]. More recently, the structures of the two main targets of OA, the protein phosphatases type 1 (PP1) and 2A (PP2A) were reported in complex with this toxin [14,15]. The availability of these structures and the discovery of new inhibitors have allowed a better understanding of the molecular basis underpinning the observed differences in protein selectivity [16,17,18], and could be the opening for the design of new drugs targeting cell signaling by protein phosphatase inhibition [19].

## 2. Dimerization of Okadaic Acid Overview

### 2.1. Complexation of Okadaic Acid

**Figure 1:**
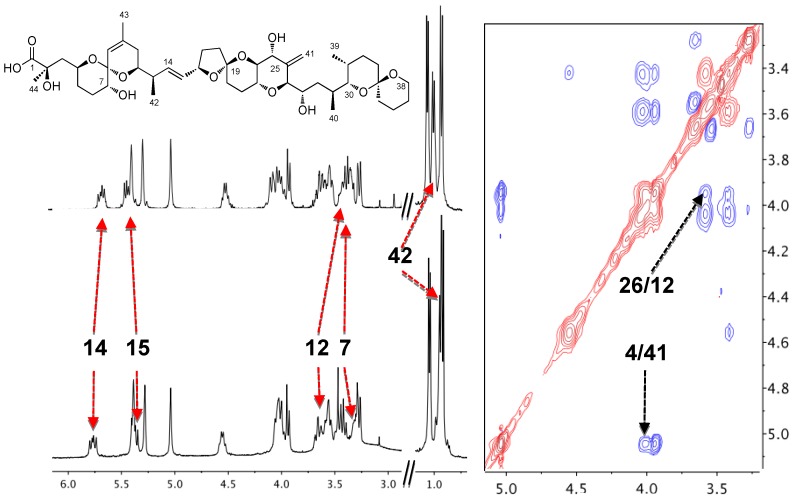
^1^H-NMR spectra of okadaic acid (upper left corner), its complex (bottom left corner) and section of the ROESY of the complex in CDCl_3_ (right).

Norte *et al*. first reported in 1991 that the toxin was usually isolated from cultures of *P. belizeanum* as a complex with an unknown ion [6]. At first glance, the main characteristic of the complexed form of okadaic acid (OAC) NMR spectra was its low resolution. Nevertheless, sample treatment with an ion chelator such as EDTA, modified the spectrum that became well resolved. In addition, signals from H-7, H-12, H-14, H-15, H-23 and Me-41 clearly changed their chemical shifts upon addition of EDTA (Figure 1). Following this opening observation, a series of experiments were carried out in order to identify the ion included in this complex [20]. The experiments consisted of the treatment of OA samples with different metal ions such as Li^+^, Na^+^, K^+^, Ca^2+^ and Mg^2+^. Those ions were selected based on the hypothesis that the genuine one should have a physiologically relevant role and must have an atomic volume that could fit into a “cavity” formed by OA units. In fact, treatment of OA with K^+^ salts resulted in ^1^H-NMR spectra that resembled those of the naturally isolated samples, giving an important clue about the ion identity.

### 2.2. Physiological Importance of the Potassium Ion

Further confirmation of the ion nature was obtained from pharmacological experiments [20]. Briefly, taking into account that OA induces smooth muscle contractions, rat uterine strips were incubated either with OA or OAC in Ca^2+^, Na^+^ or K^+^ free PSS media. The outcome was that while the physiological effect of OAC was maintained in a K^+^ free solution, the activity of OA was severely reduced, confirming the previous observations and indicating that the potassium ion was involved in the mechanism of action of this toxin.

### 2.3. Structure of the Okadaic Acid—Potassium Complex

**Figure 2:**
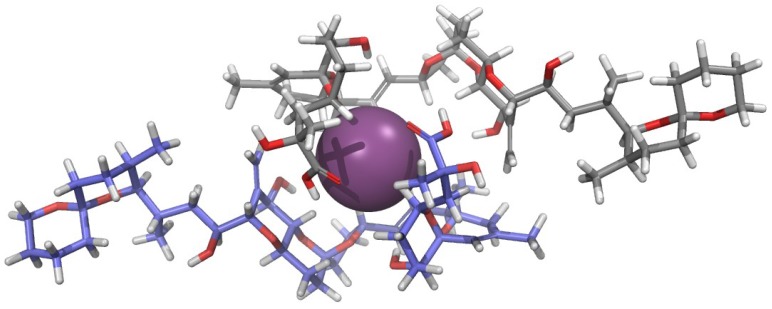
Structure proposed for the okadaic acid—potassium complex [21]. Each okadaic acid monomer is shown in a different color while K^+^ is shown as a purple sphere.

The aforementioned pharmacological results encouraged the authors to start a structural study of OAC in solution in order to explain the physiological importance of the complex [21]. Surprisingly, the NOESY spectrum of a 1 mg/mL sample of OAC recorded in CDCl_3_ did not show cross-correlated signals contrary to what had been observed for OA. This observation suggested that both species have different correlation times, and that the molecular volume of OAC was larger than that of OA. This conclusion was confirmed by measurements of translational diffusion coefficients in CDCl_3_ obtained from NMR Diffusion-Ordered SpectroscopY (DOSY). Conversion of these measurements into apparent molecular weights (M_app_) using the Stokes-Einstein equation corroborated that two units of OA formed OAC in CDCl_3_ solution. In contrast, DOSY experiments performed in water showed little differences between the two species (OA and OAC), suggesting that the monomer-dimer equilibrium in this solvent is shifted to the monomeric species. Finally, our findings were reinforced by mass spectrometry experiments, where it was observed that ESI-MS of OAC samples isolated from cultures exhibited characteristic [OA_2_ + K + H_2_O]^+^ and [OA_2_ + K]^+^ peaks, confirming that OAC was a dimer of OA and that the potassium ion was part of it. Next, measuring ^1^H-^1^H coupling constants and using distance constrains derived from the ROESY experiment (Figure 1), a molecular model of OAC was built based on Monte Carlo conformational searches. At this point, it is worth considering that one additional and very important restriction imposed to the produced model was its necessary symmetry as it was deduced from the absence of NMR signal duplication. The outcome was a model where the potassium ion-binding site was formed by three oxygen atoms attached to C-7 and C-8 of each OA monomer (Figure 2). The proposed structure therefore has a polar coordination site buried by a hydrophobic surface [21]. Thereafter, we considered the possibility that this structure could be a means of improving OA membrane permeability in accordance with the fact that the dimer was mainly found in CDCl_3_—a solvent that somehow mimics the lipid bilayer environment—and keeping in mind the bioactivity results obtained from experiments performed in tissues.

### 2.4. Dimerization as a Pathway to the Cell

**Figure 3:**
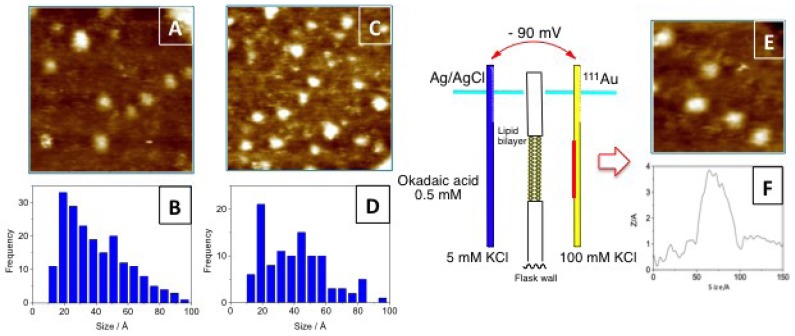
(**A**) STM image of an okadaic acid (OA) sample and (**B**) its corresponding histogram of size distribution. (**C**) and (**D**) are the corresponding figures of okadaic acid in a KCl solution. On the right side of the figure, a schematic representation of the experimental setup used to monitor the flux of OA through a lipid bilayer. (**E**) corresponds to a section of the gold plate used to monitor the experiment and (**F**) is the cross section of one spot.

In order to confirm the previous hypothesis, additional experiments were undertaken. However, we faced the problem of resolving the aggregation status of a symmetric molecule when it is interacting with membranes despite its difficult. As an alternative, we designed an experiment that used Scanning Tunneling Microscopy (STM) as a detection technique. Using this technique it is possible to measure particle sizes, therefore it was feasible to differentiate between particles formed by one or two units of OA, therefore the formation the dimers mediated by K^+^ ions was verified (Figure 3). Subsequently, a simplified model to study the transport of the toxin through a lipid bilayer was built separating two KCl aqueous solutions by a lipid bilayer [22]. One solution had the K^+^ concentration that can be found in the intracellular medium (100 mM) while the other contained 0.5 mM of OA and 5 mM of the same electrolyte (equivalent to the extracellular concentration). The detector, a gold plate, was placed into the 100 mM KCl solution but only when a −90 mV potential was applied (equivalent to the membrane potential), OA molecules circulated through the lipid bilayer and were subsequently adsorbed onto the gold plate. Analysis of the plate by STM indicated that the particle size distribution was shifted to higher values, matching those calculated for OA dimers (Figure 3).

**Figure 4:**
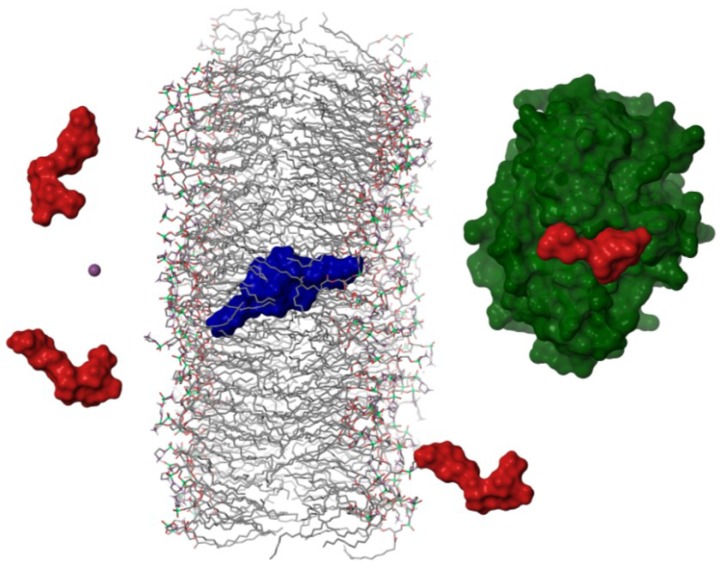
Schematic representation of the hypothetical mechanism followed by okadaic acid to cross cellular membranes. Okadaic acid monomers (in red) would bind a potassium ion (purple sphere) from the extracellular media in order to form a dimer with a hydrophobic surface (in blue), that would cross lipid bilayers allowing the toxin to bind the catalytic subunit of protein phosphatases (PP2A in green [15]).

As a consequence, we proposed that in order to move through biological membranes OA should dimerize. Once the dimer reaches the cytoplasm, the equilibrium is shifted back to the monomer, in accordance with the DOSY results carried out in water. In this way, the toxin would be able to interact as a monomer with their main cellular targets, the serine-threonine protein-phosphatases type 1 and 2A (Figure 4).

## 3. Results and Discussion

### 3.1. Higher Oligomerization States of Okadaic Acid

**Figure 5:**
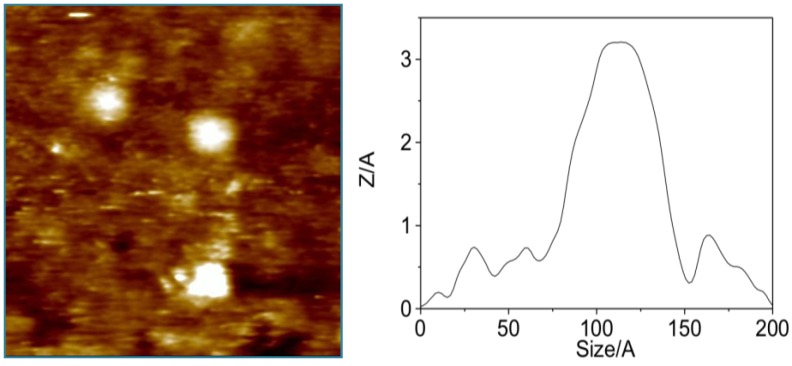
Representative STM images obtained after dipping a gold plate into an okadaic acid methanol solution. The left panel is a 20 nm × 20 nm section from an overhead viewpoint. The right panel is a cross-section corresponding to an okadaic acid tetramer obtained from the same area.

In our experience, during the isolation of OA from cultures of the dinoflagellate *Prorocentrum lima*, it is common to notice slightly different chromatographic behaviors between different batches. Nevertheless, their NMR spectra are equivalent to those reported in either the monomeric or the dimeric forms of okadaic acid. In this way, using an OA sample eluted at a different chromatographic retention time, which ^1^H NMR spectra resembled that previously reported for the dimeric form of okadaic acid, a nanospray ESI-MS spectrum was acquired. The unexpected outcome was the presence of an additional clear peak at *m/z* 3336.25. This result implies the co-existence of monomeric and dimeric species, also observed in the spectra at *m/z* 1697.4 [(OA)_2_ + H_2_O + MeOH + K]^+^ and 1649.4 [(OA)_2_ + H_2_O + Na]^+^, with higher molecular weight species that according to its molecular weight should be formed by four units of OA. As this observation cannot be considered conclusive, we looked for further experimental evidences that support the existence of tetramers in solution. NMR measurements were not adequate, because, as described previously, OAC samples in CDCl_3_ solution behave mainly as dimers, according to its diffusion coefficient. However, such measurements do not discard the existence of minor populations of monomers and/or tetramers. Considering that we have successfully used scanning tunneling microscopy (STM) to confirm the existence of dimeric forms of OA [22], we decided to follow a similar approach to confirm the existence of its tetrameric forms. In order to do this, a gold plate was dipped into a methanol solution of OA (10 mg/mL) and as expected, a gold surface mainly covered by 40–45 Å particles that correspond to OA dimers (Figure 5). It was also possible to find a small number of particles of 20–25 Å, consistent with the presence of OA monomers, and more interestingly, OA aggregates of 50–60 Å that can be rationalized by the existence of OA tetramers. Therefore, this experiment confirmed that monomer/dimer/tetramer species coexist in solution.

### 3.2. Structure of Okadaic Acid Tetramers

Once the existence of OA tetramers was confirmed, a series of NMR spectra were acquired in order to obtain structural information at an atomic level. However, no additional information was found as the observed ^1^H and ^13^C-NMR chemical shifts of the studied samples matched very well with those of the dimeric species. In addition, the ROESY experiment showed the same dipolar correlations that can be observed in the dimeric form of OAC (H-4/H-41 and H-12/H-26) that allowed us to propose a structure for the dimeric form (Figure 1, Figure 2). Moreover, the absence of new signals is indicative of the existence of a symmetric arrangement of the molecules involved. However, the structural determination of symmetric polymeric species by NMR techniques is a very difficult task. The main reason is the impossibility to differentiate between equivalent atoms precluding the use of angular or distance constrains obtained either from coupling constant or NOE measurements. Unfortunately, despite different conditions being assessed, we were not able to obtain crystals that could be use by X-ray crystallography techniques. Nevertheless, taking into account that the NMR data perfectly matches that already published for the dimer, we considered that the tetrameric form of OA should be formed by the combination of two dimers in a symmetric arrangement; in other words, it should be a dimer of dimers. Therefore, using these structural data, we have used a molecular modeling approach to propose a three-dimensional structure of the okadaic acid tetramer.

**Figure 6:**
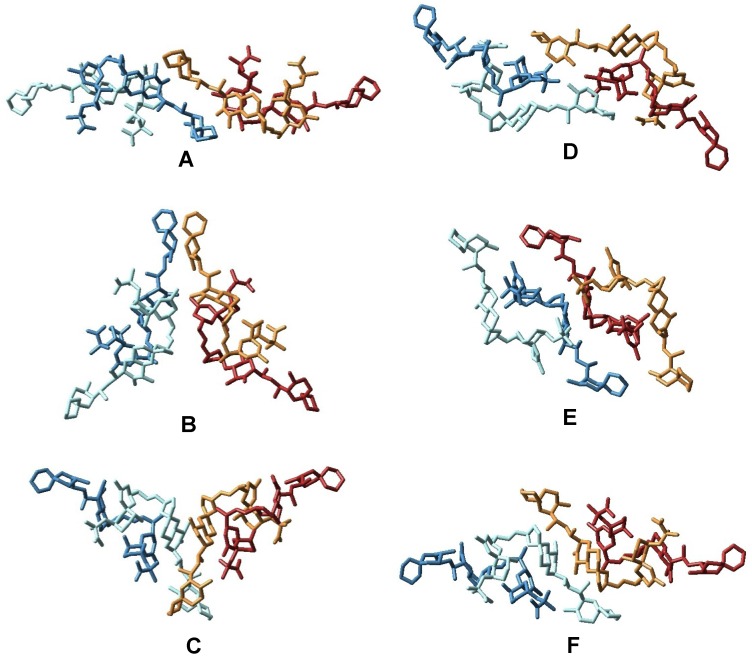
Representation of the different structural families of okadaic acid tetramers as obtained from the X-Cluster software. In this figure, OA units forming each dimer are in different shades of the same color (dark and light blue, and dark and light brown).

**Figure 7:**
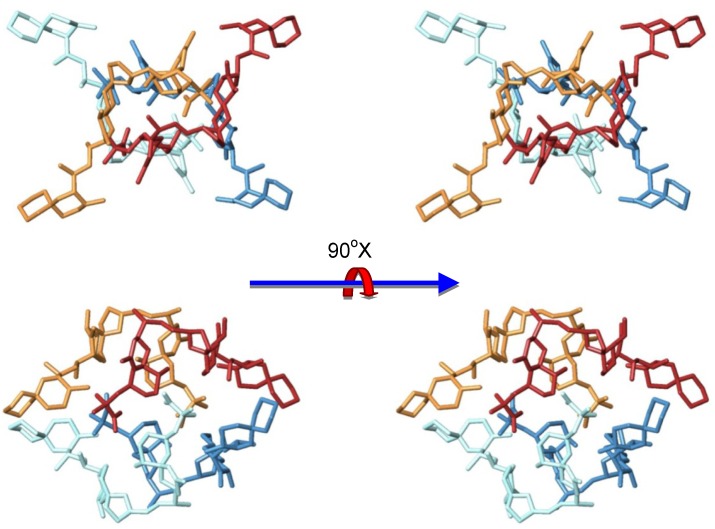
Sterereoviews of the proposed structure of the tetrameric form of okadaic acid.

Taking into account the scarce structural data available and using the ClusPro software, we explored the conformational space in order to predict the structure of the complex [23]. 20,000 different dockings between two dimers were generated and afterwards only those structures with symmetric elements were selected, resulting in 76 tetrameric structures. Afterwards, these structures were clustered using the X-Cluster software as implemented in MacroModel 8.5 and as a result, seven different structural families were obtained (Figure 6, Figure 7) [24].

After a careful analysis of these seven representative structures, we concluded that only the structure of one of them, family in Figure 7, could satisfy all previously described structural features of our sample. Certainly, only structure G keeps the C-30–C-38 moiety of each OA unit far away from each other as they are in the dimer, a characteristic that would result in very similar chemical shifts as compared to those observed for the dimer. On the other hand, in all other structures (A–F) new contacts between at least two of these C-30–C-38 fragments would be observed, resulting in important changes in their corresponding chemical shifts. In addition, structure G also maintains the hydrophilic cavity where three molecules of solvent can be fitted, according to the molecular weight found in the MS experiment *m/z* 3336.25 [(OA)_4_ + (MeOH)_3_ + Na]^+^. This model is consistent with the chemical structure: Four units of OA that are symmetrically organized and an internal cavity where three molecules of solvent can be accommodated. Finally, it should be noted that the dimensions of the selected structure corresponds quite well with the dimensions determined for the tetramer according to the cross section measured in the STM image (Figure 5).

## 4. Experimental Section

### 4.1. Spectroscopic and Spectrometric Techniques

NMR spectra were performed on a Bruker AVANCE 400 MHz instrument. ^1^H and ^13^C NMR chemical shifts are referenced to the CD_3_OD solvent peak (*δ*_H_ 7.26 ppm and *δ*_C_ 77.0 ppm) at 300 K. The compound was dissolved in 250 μL of CDCl_3_ in a Shigemi tube. Phase-sensitive ROESY spectra were measured using a mixing time of 400 ms as the intensity of the NOESY cross-peaks were close to zero at room temperature. Data were processed using Topspin or MestReC software. Mass spectra were recorded in a Thermo Finnigan LQC spectrometer, quipped with a NanoSpray source, and the sample was applied dissolved in MeOH at 50 nL/min flow. STM images (constant current mode) were taken with a Nanoscope IIE (Digital Instrument, Santa Barbara, CA) using both, commercial and electrochemically etched Pt-Ir tips. All samples were thoroughly rinsed with water, methanol and dried and under N_2_ flow for several hours before imaging. All images were taken in air at room temperature. Typical tunneling current and applied bias voltages to obtain good quality images were 0.2–0.4 nA and 0.3–0.6 V respectively at scan rates ranging 0.5–2 Hz. The STM images shown here constitute representative results. Au plates (Arrandee™) were used as substrate. These plates after a flame annealing consist of micrometer-sized Au (111) preferred oriented crystals with atomically smooth triangular terraces separated by monatomic steps in height. The height of these steps (2.4–2.6 Å) was used to calibrate the piezotube of the STM in the *z* direction. Some of the images were analyzed by using WSxM software [25]. TLC was performed on AL Si gel Merck 60 F254, and TLC plates were visualized by spraying with phosphomolybdic acid reagent and heating.

### 4.2. Prorocentrum Belizeanum Cultures

A 3 mL sample of a clonal culture (7000 cells/mL) of the dinoflagellate *Prorocentrum belizeanum* (PBMA01), originally isolated from a coral reef of La Réunion Island (Indian Ocean, France), was obtained from the IEO culture collection (Vigo, Spain), by courtesy of Mr. Santiago Fraga. Large–scale cultures of *P. belizeanum* were performed in 80 L tanks containing 40 L of seawater enriched with Guillard K medium at 23 ± 1 °C, with irradiance of 60 µE/s/m^2^ and under an 18:6 light/darkness photocycle. Cultures were incubated statically for 50 days up to a final volume of 750 L.

### 4.3. Extraction, Isolation and Purification

Due to the benthonic nature of the dinoflagellate *P. belizeanum*, most of the supernatant was easily separated by decantation and cells were harvested by centrifugation (3700× *g* for 10 min at 4 °C). The cells were sonicated and successively extracted with acetone (5 × 800 mL) and then with methanol (5 × 800 mL) to obtain two separated extracts. The methanol extract (14.95 g) was chromatographed on a Sephadex LH-20 column (60 × 350 mm) eluted with methanol and then fractionated using a Lobar LiChroprep RP8 column (25 × 310 mm) eluted with CH_3_OH/H_2_O (7:3). The selected fraction (0.05 g) was subjected to gel filtration on a Sephadex LH-20 column (10 × 150 mm) eluted with CH_3_OH/CHCl_3_ (1:2). Final purification of okadaic acid (50 mg) was achieved by semi preparative reversed-phase HPLC (XTerra Prep C18 10 µm, 19 × 150 mm, 1.0 mL/min flux) using an isocratic elution of CH_3_OH/H_2_O (17:3).

### 4.4. Preparation of STM Gold Plate

The gold plated was dipped in a 10 mg/mL OA (12.4 mM) methanol solution for 5 min. This was washed with methanol, dried and analyzed by STM microscopy at 1 nm/div.

## 5. Conclusions

In this article, we have reviewed the existing knowledge about the dimerization of okadaic acid, the implication of metal ions in this process and their possible physiological implications. The elucidation of the okadaic acid dimer structure in solution was also reviewed. The proposed structure shows a hydrophobic surface that would increase membrane permeability of the toxin, providing structural clues about the physiological importance of dimerization as well as about the role of potassium ion observed in the *in vivo* action of OA. The exact role of the dimeric species of OA is uncertain but our results indicate that it is vital for the membrane transport of the toxin. Although self-association is not a common phenomenon in marine toxins, it has been observed in brevetoxin-B where transmembrane self-assembly was proposed to explain cation transport via a “channel-like” mechanism and in palytoxin where a dimeric structure has been recently determined by means of SAXS and NMR spectroscopy, although no physiological consequences have been proposed for the latter [26,27,28]. In addition, although the results are not conclusive, our mass spectrometry, NMR spectroscopy and STM imaging experiments suggest, that the marine polyether toxin okadaic acid self-associates to form a tetramer when it is in high concentration coexisting with monomers and dimers of the toxin. Finally, utilizing molecular modeling techniques, we propose a structure for the tetrameric form of okadaic acid.
